# Focus on craniomaxillofacial injuries in trauma patients

**DOI:** 10.1007/s00068-022-02036-4

**Published:** 2022-08-08

**Authors:** Shahram Ghanaati

**Affiliations:** Department for Oral, Cranio-Maxillofacial and Facial Plastic Surgery, University Hospital Frankfurt, Theodor Stern-Kai 7, 60590 Frankfurt, Hessen Germany

This CMF volume of European Journal of Trauma and Emergency Surgery presents a very good symbiosis of making the maxillofacial trauma pattern and their treatment concepts understandable on a clinical relevant basis for everyone involved in managing trauma-based injuries.

Maxillofacial trauma is widely present all over the globe. Its management needs experts in maxillofacial fields. However, a large number of these trauma patterns is associated with additional injuries such as neurosurgical injuries and/or trauma surgeries of the extremities.

In the present volume care has been given to choose manuscripts clearly showing, that the cause of maxillofacial injuries can regionally differ from that what we know from injuries within European countries Al‐Hassani et al*.* [1]. Thus, with different cause of injuries, different trauma management is necessary accompanying with region-related morbidity and mortality. In comparison to that, the paper of Muhlenfeld et al*.* [2] highlights the maxillofacial injury pattern and cause in a modern European country trauma center, reflecting the necessity of an interdisciplinary approach between maxillofacial surgeons, trauma surgeons as well as neurosurgeons. Another very interesting aspect is that the majority of maxillofacial trauma-based injuries is the affection of the maxilla along a further affection of the bony parts, which build the walls of the orbita as shown in the paper of Girish Rao et al*.* [3].

The data of the articles considered underline the importance of road and traffic accidents as a major source for the incidence of the maxillofacial injuries, reflecting the differences in regional awareness of using cars and bicycles as daily vehicles. They underline as well, that midfacial injury patterns seem to be the most common injuries independent of the geographical localization.

Another important aspect of this volume is the focus on horse-related injuries. The work by Stier et al*.* [4] underlines the necessity of a proper equipment and clearly shows the interdependency regarding the severity of the injury intensity. Finally, the manuscript by Rozema et al. [5] brings evidence, that a systematic approach for examination is necessary in managing maxillofacial traumas in order to achieve the best possible outcome in making a decision, while reducing the number of unnecessary and time consuming diagnostic tools and steps. I am sure, that this volume is an effective scientific tool for colleagues involved in trauma management to enhance their knowledge in managing facial traumas and trauma-related morbidity. The understanding of the trauma pattern of CMF injuries and their indication-based specific surgical management facilitates the treatment conception for both CMF and other specialists of any trauma and emergency surgery-treating center. Considering that CMF traumas represent around 30% of all admitted trauma cases underlines that in many cases interdisciplinary surgical conception by specialists such as trauma surgeons, neurosurgeons as well as CMF-surgeons is necessary.

Based on the present volume I sincerely hope that the importance of understanding the origin and treatment management of CMF injuries in trauma arises.
